# Mpox in endemic regions in Nigeria: awareness, knowledge, and willingness to accept the mpox vaccine

**DOI:** 10.11604/pamj.supp.2025.50.1.44293

**Published:** 2025-06-03

**Authors:** Isaac Iyinoluwa Olufadewa, Miracle Ayomikun Adesina, Ruth Ifeoluwa Oladele, Toluwase Ayobola Olufadewa, Evaezi Okpokoro, Oluwafemi Babatunde Daodu, Fehintola Ige, Sylvia Adebajo, Ehimario Uche Igumbor, Moyinoluwa Joshua Oladoye, Joseph Ojonugwa Shaibu, Dimie Ogoina, Rosemary Ajuma Audu

**Affiliations:** 1 Centre for One Health and Zoonotic Diseases, Slum and Rural Health Initiative, Ibadan, Nigeria,; 2 International Research Centre of Excellence, Institute of Human Virology, Nigeria, Abuja, Nigeria,; 3 Department of Veterinary Microbiology, Virology Unit, Faculty of Veterinary Medicine, University of Ilorin, Ilorin, Kwara State, Nigeria,; 4 Centre for Human Virology and Genomics, Nigerian Institute of Medical Research, Yaba, Lagos State, Nigeria,; 5 Centre for International Health, Education, and Biosecurity (CIHEB), Maryland Global Initiatives Corporation (MGIC), University of Maryland, Abuja, Nigeria,; 6 Centre for Infectious Diseases Research, Nigerian Institute of Medical Research, Yaba, Lagos State, Nigeria,; 7 School of Health Systems and Public Health, University of Pretoria, Pretoria, South Africa,; 8 Department of Internal Medicine, Infectious Diseases Unit, Niger Delta University Teaching Hospital, Bayelsa, Nigeria

**Keywords:** Knowledge, awareness, mpox vaccine, mpox virus, endemic, awareness, Nigeria

## Abstract

**Introduction:**

Nigeria ranks second in Africa for the highest number of mpox cases. This study aimed to evaluate the knowledge and awareness of mpox among Nigerians living in endemic regions and their willingness to accept the mpox vaccine when available.

**Methods:**

we conducted a cross-sectional study using a multi-stage sampling technique. Data was collected from eligible individuals in Bayelsa, Delta, Lagos, and Rivers, Nigeria, between September 1 and November 30, 2023, using a standardized structured questionnaire. Descriptive analysis was conducted, and inferential analyses were performed using binary logistic regression (p < 0.05).

**Results:**

five hundred and twenty-four (524) persons with a mean age of 33.9 ± 10.4 years participated in this study. Fifty-eight percent (58%) were aware of mpox, and 15.5% of participants had heard about the mpox vaccine. Participants from Delta State were 70 percent less likely, while those from Lagos and Rivers States were 2.5 times and 1.04 times, respectively (OR= 2.48, p=0.012; OR= 1.04, p=0.89) more likely to receive the mpox vaccine when compared with participants from Bayelsa State. Eighty-four (84.5%) of respondents were unwilling to take the vaccine if they had to pay for it.

**Conclusion:**

although many people were aware of the mpox infection, only a few people were aware of the vaccine. Health intervention programs to improve knowledge of mpox and increase the uptake of mpox vaccines should be co-designed with community stakeholders, while mpox vaccines, when available, should be made accessible at subsidized or at no cost to Nigerians to improve uptake.

## Introduction

Mpox (formerly monkeypox) is a viral zoonotic disease caused by the mpox virus (MPXV) transmissible from human to human, human to animal, and/or animal to human [1]. Mpox virus belongs to the orthopoxvirus genera and the Poxviridae family, and it was first identified in 1958 among laboratory monkeys. The first human case of mpox was identified in 1970 in an infant in the Democratic Republic of Congo [2]. In July 2022, the World Health Organization declared mpox a public health emergency of international concern, following a global disease outbreak in May 2022 [3]. As of 30 April 2024, a total of 974,208 confirmed cases and 186 deaths were reported globally across 117 countries [WHO 2024], including 3,094 cases and 24 deaths from endemic regions of Africa. Nigeria has the second-highest number of cases (858) in Africa, next to the DRC [4].

Since the atypical global outbreak in 2022, several studies have explored the causes, clinical characteristics, transmission routes, and outcomes of mpox globally [1,5-8]. Transmission of MPXV is mainly through close contact (skin-to-skin or mouth-to-skin) with someone who is infected with the virus or indirectly via contaminated objects [2]. While there is no approved specific treatment for mpox, antiviral medications like cidofovir, brincidofovir, tecovirimat, and vaccinia immune globulin have been administered under compassionate use or emergency use authorization. The evidence for the clinical use of these drugs was based on findings from research, mainly from the variola virus, the cause of smallpox, which belongs to the same orthopoxvirus genus as MPXV [1,9]. Also, recent research has focused on developing and approving a vaccine to prevent the spread of mpox, with the non-replicating smallpox vaccine (the Modified Vaccinia Ankara-Bavarian Nordic- MVA-BN) being approved as a temporary vaccine to protect individuals at risk of mpox [10].

Findings from research studies have shown that strengthening awareness and surveillance of mpox is critical to controlling the spread of an outbreak [2,11]. While vaccination has proved to be successful, safe, and effective in mitigating several infectious disease outbreaks [2], it is often accompanied by vaccine hesitancy, which is a global threat [4] that may hamper the prevention and control of the mpox disease outbreak.

Studies to assess awareness and knowledge of mpox are sparse in Nigeria. Awoyomi et al. conducted an online cross-sectional study that assessed the perceptions and knowledge of 1544 healthcare workers, academics, and students in tertiary institutions [12]. Another study conducted by Ajayi et al. assessed the knowledge and awareness of mpox infection among 316 healthcare workers in one state (Ekiti state) in Nigeria [13]. Both studies were focused on healthcare workers and/or those in tertiary institutions, rather than the general population, a limitation our study addresses. Furthermore, to our knowledge, no study has been conducted to determine the willingness to take the mpox vaccine in Nigeria and sub-Saharan Africa. This study explored the awareness, knowledge of mpox, and willingness to receive the mpox vaccine in four states (Bayelsa, Delta, Lagos, and Rivers states) endemic with mpox in Nigeria. This study assessed the factors influencing the awareness of mpox and the mpox vaccine, as well as factors influencing participants´ willingness to receive the Mpox vaccine. Findings from this study would be useful for informing public health programs, designing interventions to prevent and control the spread of mpox, as well as increasing mpox vaccine uptake when it becomes available in Nigeria.

## Methods

**Study setting and study participants:** this study was conducted in four states in Nigeria - Bayelsa, Delta, Lagos, and Rivers. The four states were selected purposively because they had the highest number of mpox cases during the 2022 outbreak [14]. All four states are subdivided into three senatorial zones. Each senatorial zone is further subdivided into local government areas (LGAs), and each local government area is divided into political wards.

**Study design and sampling:** this was a cross-sectional study using a multi-stage sampling technique. Residents in communities and healthcare facilities in selected political wards across the four states who were aged 18 and above and who had resided in their current location for at least six months were randomly selected. Those who lived in prisons and military barracks (institutionalized settings) were excluded. In each of the four states (Bayelsa, Delta, Lagos, and Rivers) where this study was conducted, two local government areas (LGAs) were selected using a systematic sampling technique. First, the list of all LGAs within each state was obtained from official government records. A simple random sampling technique was then employed to select two LGAs per state. Each eligible LGA was assigned a unique identification number, and a fish-bowl sampling technique was used to ensure unbiased selection. After the initial selection, the chosen LGAs were reviewed to confirm their feasibility for the study. In cases where logistical challenges, security concerns, or other practical constraints were identified, an alternative LGA was randomly selected from the remaining eligible list.

To identify the healthcare facilities, a comprehensive list of registered health institutions within the selected LGA was obtained from the local health authorities. These facilities were categorized into public and private healthcare providers. One facility from each category was selected using a simple random sampling technique. Household selection was conducted within a designated political ward in each LGA. The process followed a systematic random sampling using the list of households from the political wards. The total number of households in the ward was divided by 25 to determine a sampling interval. A random starting point was selected within the first interval, and subsequent households were chosen at fixed intervals (e.g., every nth household) until the required sample size was reached.

**Data collection:** data was collected using a standardized questionnaire that was administered by trained research assistants using Research Electronic Data Capture (REDCap). Data were collected from the study participants between September 1 and November 30, 2023. The questionnaire comprised three categories of questions: (1) sociodemographic characteristics; (2) knowledge and awareness of mpox and awareness about the mpox vaccine; and (3) willingness to receive the mpox vaccine. Items of the questionnaire were developed from a review of quantitative and qualitative published literature [15-19] and were assessed for their content validity by a team of infectious diseases experts and researchers. Afterwards, the questionnaire was pre-tested on a random sample of 25 individuals from communities and healthcare facilities to ensure the clarity of the questions and interpretation of responses. The independent variables included sociodemographic characteristics, which were gender, age, state, place of residence, occupation, and their highest level of education. Dependent variables included their level of awareness and knowledge of the mpox virus infection and their willingness to take the mpox vaccine.

**Awareness of mpox and mpox vaccine:** the awareness of mpox was evaluated by asking the question: *have you ever heard about mpox?* (yes/no). Participants who responded “yes” were recorded as being aware of mpox. The awareness of the mpox vaccine was evaluated by asking the question: *have you heard about the mpox vaccine?* (yes/no). Participants who responded “yes” were recorded as being aware of the mpox vaccine.

**Knowledge of mpox:** the knowledge of mpox was measured by the following four questions: *“do you know anyone infected or suspected of having Monkeypox?´*; *“Can Monkeypox be transmitted to humans through bites and scratches from infected animals”; “Can smallpox vaccine protect people from monkeypox”; “Do you know any facility where Monkeypox can be diagnosed or treated?”*. The responses were “yes”, “no”, “not sure/undecided”. This was categorized into, “yes” and “no” (which included “No” and “not sure/undecided”).

**Willingness to receive the mpox vaccine:** participants´ willingness to receive the mpox vaccine was evaluated using five questions: “I would be willing to receive the monkeypox vaccine”; *“I would be willing to take the monkeypox vaccine even if I have to pay for it”; “I would recommend my friends and relatives to receive the monkeypox vaccine”; “I would be willing to take the monkeypox vaccine if it is manufactured in Nigeria”; “I would support the promotion of the monkeypox vaccine in my community”*. The responses were “yes”, “no”, and “not sure/undecided”. This was categorized into positive responses (“yes”), which were scored ‘1’, and negative responses (which included “no” and “not sure/undecided”), which were scored ´0´ (with a maximum score of 5 and minimum score of 0 points). Respondents were categorized into “more willing” if they scored 3 points or above and “less willing” if they scored 2 points or below. The questionnaire showed good reliability by a Cronbach´s Alpha internal consistency coefficient of ≥ 0.77.

**Statistical analysis:** descriptive analysis was conducted using appropriate statistics for the sociodemographic characteristics, such as frequency, percentage, mean, median, and interquartile range. Inferential analyses were performed using binary logistic regression. We determined associations between selected socio-demographic factors (independent variables) and their awareness of the mpox virus (dependent variable). We also conducted a similar inferential statistical analysis between sociodemographic factors (independent variable) and their willingness to receive the mpox vaccine (dependent variable) as dichotomized above. The results of all logistics regression are reported as odds ratios alongside their p-value (significance level), which was set at 0.05. Data were analyzed using the Statistical Package for Social Sciences (SPSS) version 26.

**Ethical consideration:** ethical approval was obtained from the National Ethics and Health Research Committee (NHREC/01/01/2007) as part study entitled “Characterizing transmission dynamics and evaluating medical countermeasures to inform clinical and public health response in Canada and Nigeria (CAMP Study). Before the data collection, approval was also sought from the community heads and the healthcare facilities. The aims of the study were explained to the study participants, and they were assured of confidentiality and autonomy. Written informed consent was obtained from all participants.

## Results

Five hundred and twenty-four (524) respondents participated in this study from Bayelsa (24.4%), Delta (25.9%), Lagos (25%), and Rivers (24.6%) out of the sample population of 588, giving an overall response rate of 89.1%. The mean age of the participants was 33.9 ± 10.41 years, with more than half of the respondents being female (56.3%), living in urban areas (64.1%), and being Christians (89.7%). Secondary education was the highest educational attainment for most participants in Bayelsa (40.6%), Delta (67%), and Rivers (54.3%), whereas a majority (76.3%) in Lagos attained tertiary education (Table 1).

**Table 1:** sociodemographic data of participants by state

Socio-demographic characteristics	States				
	Bayelsa N= 128	Delta N=136	Lagos N=131	Rivers N=129	Total N=524
	N (%)	N (%)	N (%)	N (%)	N (%)
**Age group**					
18-25	25 (19.5%)	45 (33.1%)	27 (20.6%)	38 (29.5%)	135 (25.8%)
26-35	34 (26.6%)	41 (30.1%)	45 (34.4%)	55 (42.6%)	175 (33.4%)
36-45	35 (27.3%)	42 (30.9%)	36 (27.5%)	28 (21.7%)	141 (26.9%)
46-55	22 (17.2%)	6 (4.4%)	17 (13%)	8 (6.2%)	53 (10.1%)
56-65	12 (9.4%)	2 (1.5%)	6 (4.6%)	0 (0%)	20 (3.8%)
	**Mean+SD**	33.93+10.41			
	**Median**	33			
**Gender**					
Male	62 (48.4%)	61 (44.9%)	54 (41.2%)	52 (40.3%)	229 (43.7%)
Female	66 (51.6%)	75 (55.1%)	77 (58.8%)	77 (59.7%)	295 (56.3%)
**Place of residence**					
Urban	75 (58.6%)	70 (51.5%)	127 (96.9%)	64 (49.6%)	336 (64.1%)
Rural	53 (41.4%)	66 (48.5%)	4 (3.1%)	65 (50.4%)	188 (35.9%)
**Occupation**					
Artisan	12 (9.4%)	26 (19.1%)	2 (1.5%)	20 (15.5%)	60 (11.5%)
Civil service	25 (19.5%)	4 (2.9%)	27 (20.6%)	10 (7.8%)	66 (12.6%)
Self-employed	21 (16.4%)	57 (41.9%)	33 (25.2%)	33 (25.6%)	144 (27.5%)
Student	14 (10.9%)	14 (10.3%)	27 (20.6%)	21 (16.3%)	76 (14.5%)
Trader	31 (24.2%)	7 (5.1%)	24 (18.3%)	29 (22.5%)	91 (17.4%)
Unemployed	15 (11.7%)	11 (8.1%)	1 (0.8%)	11 (8.5%)	38 (7.3%)
Others	10 (7.8%)	17 (12.5%)	17 (13%)	5 (3.9%)	49 (9.4%)
**Highest level of education**					
None	14 (10.9%)	0 (0%)	0 (0%)	1 (0.8%)	15 (2.9%)
Primary	29 (22.7%)	4 (2.9%)	1 (0.8%)	21 (16.3%)	55 (10.5%)
Secondary	52 (40.6%)	91 (66.9%)	30 (22.9%)	70 (54.3%)	243 (46.4%)
Tertiary	33 (25.8%)	41 (30.1%)	100 (76.3%)	37 (28.7%)	211 (40.3%)
**Religion**					
Islam	1 (0.8%)	1 (0.7%)	34 (26%)	0 (0%)	36 (6.9%)
Christianity	112 (87.5%)	135 (99.3%)	96 (73.3%)	127 (98.4%)	470 (89.7%)
Traditional	15 (11.7%)	0 (0%)	1 (0.8%)	2 (1.6%)	18 (3.4%)

**Awareness and knowledge of mpox and mpox vaccine:** fifty-eight percent (58%) of the respondents were aware of mpox. However, more respondents in Delta (61.8%), Lagos (66.4%), and Rivers (58.9%) had heard about mpox compared to Bayelsa (44.5%). Notably, at least one participant from Delta, Lagos, and Rivers reported knowing someone who was or suspected to be infected with mpox. More than half of the participants in Lagos (60.3%) and Rivers (52%) correctly reported that mpox could be transmitted to humans through bites and scratches from infected animals. The majority of the participants (83.6%), (95.6%), (76.3%), and (93.8%) from Bayelsa, Delta, Lagos, and Rivers, respectively, did not know that the smallpox vaccine has been used for mpox prevention (Table 2). On awareness about the mpox vaccine, 15.5% of all the participants had ever heard about the mpox vaccine, with 13.2%, 22.1%, and 20.9% in Bayelsa, Delta, Lagos, and Rivers respectively indicating their awareness about the vaccine.

**Table 2:** awareness and knowledge of mpox and mpox vaccine among participants by state

Questions		States				
		Bayelsa	Delta	Lagos	Rivers	Total
		N= 128	N=136	N=131	N=129	N=524
Have you ever heard about mpox	Yes (%)	57(44.5%)	84(61.8%)	87(66.4%)	76(58.9%)	304(58%)
	No (%)	71(55.5%)	52(38.2%)	44(33.6%)	53(41.1%)	220 (42%)
Do you know anyone infected or suspected of having mpox?	Yes (%)	0(0%)	2(1.5%)	1(0.8%)	1(0.8%)	4(0.8%)
	No (%)	128(100%)	134(98.5%)	130(99.2%)	128(99.2%)	520(99.2%)
Mpox can be transmitted to humans through bites and scratches from infected animals	Yes (%)	56(43.8%)	47(34.6%)	79(60.3%)	67(51.9%)	249(47.5%)
	No (%)	72(56.2%)	89(65.4%)	52(39.7%)	62(48.1%)	275(52.5%)
The smallpox vaccine can protect people from mpox	Yes (%)	21(16.4%)	6(4.4%)	31(23.7%)	8(6.2%)	66(12.6%)
	No (%)	107(83.6%)	130(95.6%)	100(76.3%)	121(93.8%)	458(87.4%)
Do you know of any facility where mpox can be diagnosed or treated?	Yes (%)	5(3.1%)	4(2.7%)	4(2.9%)	4(3.1%)	14(2.7%)
	No (%)	124(96.9%)	132(97.3%)	127(97.1%)	125(96.9%)	510(97.3%)
Have you heard about mpox vaccine?	Yes (%)	7(5.5%)	18(13.2%)	29(22.1%)	27(20.9%)	81(15.5%)
	No (%)	121(94.5%)	118(86.8%)	102(77.9%)	102(79.1%)	443(84.5%)

**Figure 1:**
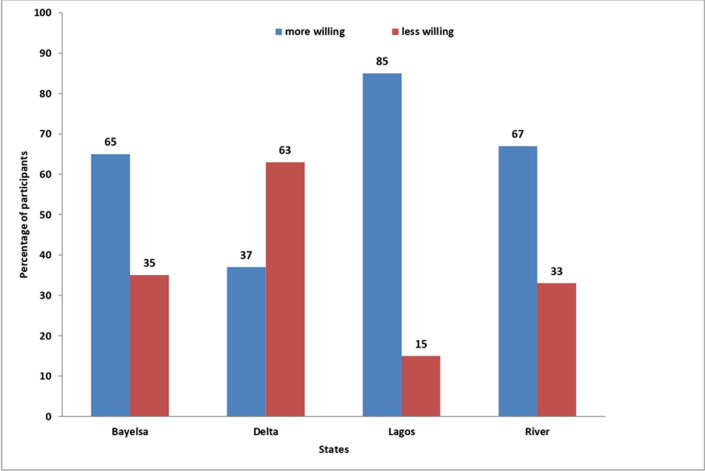
willingness of participants to receive the mpox vaccine across four states in Nigeria (Bayelsa, Delta, Lagos, Rivers)

**Willingness to receive the mpox vaccine when available:** overall, two-thirds (66.4%) of the participants were willing to receive the mpox vaccine when available. Across the states, willingness to receive the mpox vaccine varied between 38% and 85% (85% in Lagos, 74% in Bayelsa, 70% in Lagos, and 38% in Delta). Furthermore, the majority of the participants from Bayelsa (81, 63.3%), Lagos (120, 91.6%), and Rivers (90, 69.8%) stated that they would recommend their friends and relatives to receive the mpox vaccine, whereas in Delta state, about 58.2% disagreed. Table 3 presents the gender-disaggregated data on the willingness of participants to receive the mpox vaccine by gender. The majority from Bayelsa (77, 60.2%), Delta (109, 80.1%), Lagos (74, 56.5%), and Rivers (109, 84.5%) stated that they would not take the mpox vaccine if they had to pay for it. More than two-thirds (92, 70.2%) of the participants from Lagos indicated their willingness to receive the mpox vaccine if it was manufactured in Nigeria, while more than half of the participants from Bayelsa (74, 57.8%) and Rivers (76, 58.9%), with the majority from Delta (96, 70.6%), disagree on receiving the vaccine if it was manufactured in Nigeria. The majority of participants from Bayelsa (79, 61.7%), Lagos (122, 93.1%), and Rivers (104, 80.6%) indicated their willingness to promote the mpox vaccine in their community, except in Delta state where the majority (85, 62.5%) of the participants disagree (Figure 1).

**Table 3:** willingness of participants to receive monkeypox vaccine by gender

Willingness of participants		Gender		
		Male	Female	Total
		N= 229	N=295	N=524
I would be willing to receive the monkeypox vaccine	Yes (%)	153(66.8%)	195(66.1%)	348(66.4%)
	No (%)	76(33.2%)	100(33.9%)	176(33.6%)
I would be willing to take the monkeypox vaccine even if I have to pay for it	Yes (%)	64(27.9%)	91(30.8%)	155(29.6%)
	No (%)	165(72.1%)	204(69.2%)	369(70.4%)
I would recommend that my friends and relatives receive the monkeypox vaccine	Yes (%)	158(69%)	189(64.1%)	347(66.2%)
	No (%)	71(31%)	106(35.9%)	177(33.8%)
I would be willing to take the monkeypox vaccine if it is manufactured in Nigeria	Yes (%)	104(45.4%)	129(43.7%)	233(44.5%)
	No (%)	125(54.6%)	166(56.3%)	291(55.5%)
I would support the promotion of the monkeypox vaccine in my community	Yes (%)	159(69.4%)	197(66.8%)	356(67.9%)
	No (%)	70(30.6%)	98(33.2%)	168(32.1%)

**Factors influencing the awareness of mpox:** compared to participants from Bayelsa state, those from Delta, Lagos, and Rivers were less likely to be aware of mpox (OR= 0.47, p= 0.016; OR= 0.87, p=0.675; OR= 0.48, p= 0.013p). The result was not significant for other variables such as age, gender, marital status, and occupation.

**Factors influencing the level of willingness to receive mpox vaccine:** participants from Delta state was less willing to receive the mpox vaccine, while participants from Lagos state and River´s state are 2.5 times and 1.04 times more willing to receive the mpox vaccine, respectively, than participants from Bayelsa state (OR= 0.30, p<0.001; OR= 2.48, p=0.012; OR= 1.04, p=0.89). Participants who were married were more willing to receive the mpox vaccine compared with single participants, which was statistically significant (OR= 1.50, p= 0.032). Participants who are civil servants are 2.2 times more likely to be willing to accept the mpox vaccine than artisans (OR=2.209, p=0.157). The self-employed, students, traders, unemployed and others are 34%, 61%, 57%, 66% and 52% less likely to be willing to accept the mpox vaccine respectively than those artisans (OR= 0.659, p=0.246; OR= 0.393, p = 0.050; OR= 0.530, p= 0.108; OR= 0.337, p= 0.026; OR= 0.484, p= 0.115).

## Discussion

This study examined the knowledge and awareness of mpox and willingness to receive the mpox vaccine among selected individuals in four Nigerian states (Bayelsa, Delta, Lagos, and Rivers). We found that the majority of the participants were aware of infection, while only a few participants were aware of the mpox vaccine. Fewer participants (15.5%) were willing to receive the mpox vaccines when asked to pay for them. Our findings revealed that the number of participants aware of mpox varied across the four states, with Bayelsa accounting for the lowest number of participants aware of mpox of awareness. This is probably because the study was conducted in high prevalence states in Nigeria [14].

In a similar study conducted by Meo et al. among the general population in Saudi Arabia, the authors reported a satisfactory knowledge of mpox among the study participants, which was attributed to high levels of endorsements of public perceptions about the disease [19]. Another study conducted by Wogu et al. on media reportage of mpox in southern Nigeria showed that the media failed to create comprehensive awareness to educate the public on mpox, and individuals had to learn about the disease from their friends and other institutions [20]. This could also be pointers that social media, which is a common source of information for most people, should not only contain news creating awareness about a disease, but also enough information to ensure that knowledge about the disease is readily available and in simple language.

The majority of the participants were willing to receive the vaccine, which could be a strong indicator that Nigeria is winning the fight against vaccine hesitancy in its population groups. In contrast to several studies [21,22] that have reported a great gender disparity in willingness to receive vaccines, this study showed no such disparity, as both females and males were willing to receive the vaccine. Participants who were very educated and who were civil servants were more willing to receive the vaccine compared to those who were uneducated and self-employed. Also, urban residents were more willing to receive the vaccine when compared to those living in rural areas. These findings are similar to studies among different population groups conducted to assess the impact of socio-demographic factors on the uptake of vaccines [6,23].

A major concern raised by participants that could affect their willingness to receive the vaccine was whether they had to pay out of their pocket for the vaccine. This finding is unsurprising as most Nigerians are poor [24], and only 3% of the population have health insurance [25]. Previous research has also shown that people are often reluctant to bear out-of-pocket payment costs for vaccines despite the awareness of the disease and its risks [26]. Findings from our study on the knowledge of mpox among the population showed that many do not know about the disease and this may affect their willingness to pay to receive the vaccine.

To enhance the uptake of the mpox vaccine when it becomes available, health education efforts on mpox diseases and the vaccines must be offered in a simple-to-understand language, the vaccine should be made free, and primary health care centers should be utilized as vaccination centers, as they are closer to people´s residences. Ayorinde et al. emphasized the importance of community strategies to promote uptake of the mpox vaccine [27]. This study highlights the need to devise targeted health information campaigns to increase the knowledge of people about mpox, particularly in Bayelsa and Delta, where the knowledge seems to be the lowest. The poor knowledge of mpox in Delta may be attributed to the low level of education and the self-employment status of many of the study participants. Also, contextualization of mpox information is important, such as the use of local languages to educate community members. Also, measures to reduce vaccine hesitancy, especially in Bayelsa and Delta states, have to be implemented by building trust in community members about the safety of the vaccines.

To our knowledge, this study is the first to provide evidence of the willingness of Nigerians to receive the mpox vaccine when it is available. Furthermore, this study reported on mpox awareness and knowledge from relatively high mpox-prevalent states in Nigeria, thereby providing the necessary evidence to inform state-level and national-level campaigns in Nigeria. Our study focused on endemic regions in Nigeria with the most burden of mpox in the most recent outbreak (Bayelsa, Delta, Lagos, and Rivers States), which limits its generalizability to the whole Nigerian population. Future studies should endeavor to employ a larger sample size and a more geographically diverse sample across the six geopolitical zones of Nigeria.

## Conclusion

Findings from this study present disparities in the knowledge and awareness of mpox and the mpox vaccine, as well as the willingness to receive the mpox vaccine across different geographical locations. This may imply that health intervention programs to improve knowledge of mpox and increase the uptake of mpox vaccines have to be increased, as well as co-designed with community stakeholders and members in various geographical locations. Engagement with community stakeholders and members is necessary to identify the barriers and facilitators and design appropriate responses to address these barriers.

### What is known about this topic

- Knowledge and awareness of mpox infection among healthcare workers in Nigeria have been reported;
- Recent research has focused on developing and approving a vaccine to prevent the spread of mpox, with the non-replicating smallpox vaccine (the Modified Vaccinia Ankara-Bavarian Nordic- MVA-BN) being approved as a temporary vaccine to protect individuals at risk of mpox;
- Vaccine hesitancy is recognized as a global threat.

### What this study adds

- This study adds to the body of research by examining the knowledge and awareness of mpox infection among a general population group in four states in Nigeria, where mpox is endemic;
- This study also explores the willingness of Nigerians to receive the mpox vaccine when it is available.
